# Semi-quantitative and visual assay of copper ions by fluorescent test paper constructed with dual-emission carbon dots†

**DOI:** 10.1039/c8ra00917a

**Published:** 2018-04-03

**Authors:** Yifan Wang, Mian Wu, Shaoming Yu, Changlong Jiang

**Affiliations:** School of Chemistry and Chemical Engineering, Hefei University of Technology Hefei Anhui 230009 China shmyu@hfut.edu.cn; Institute of Intelligent Machines, Chinese Academy of Sciences Hefei Anhui 230031 China cljiang@iim.ac.cn; State Key Laboratory of Transducer Technology, Chinese Academy of Sciences Hefei Anhui 230031 China cljiang@iim.ac.cn

## Abstract

A novel, simple and effective dual-emissive fluorescent probe for the sensitive and selective detection of Cu(ii) has been developed by mixing blue carbon dots and orange carbon dots, with a sensitive detection limit of 7.31 nM. The blue fluorescence can be selectively quenched by Cu(ii), while the orange fluorescence is a internal reference, resulting in a distinguishable fluorescence color change from blue to orange under a UV lamp. Meanwhile, its as-prepared text paper provides a convenient and simple approach for the visual detection of Cu(ii) and successfully applied in real water samples, with a dose-discerning ability as low as 50 nM. The methodology reported here opens a novel pathway toward the real applications of fluorescent test papers.

## Abstract 2

A ratiometric fluorescence nanosensor has been developed by mixing blue fluorescent carbon dots and orange fluorescent carbon dots for the detection of copper ions.

## Graphical abstract

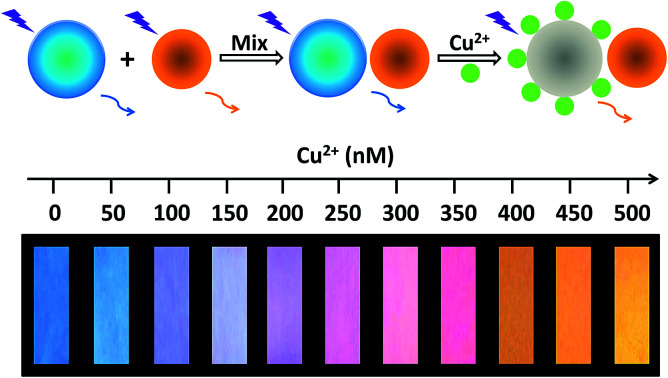

## 1. Introduction

In addition to zinc and iron, copper is the third trace metal element that is necessary for human health. Copper ions (Cu(ii)), as heavy metal ions and essential components of many enzyme systems, play an important role in some physiological and pathological processes, such as serving as a significant catalytic cofactor for the synthesis of collagen, elastin and hemoglobin.^1–3^ An excess Cu(ii) concentration may become toxic to living organisms and induce damage to the kidneys, liver and the central nervous system. And the widespread use of Cu(ii) in industry and agriculture can also lead to serious copper pollution in water and soil.^4,5^ Meanwhile, the deficiency of Cu(ii) can lead to many diseases such as bone abnormalities and anemia.^6^ The maximum allowable level of Cu(ii) in drinking water, defined by United States Environmental Protection Agency (EPA), is 20 μM.^7^ Therefore, it is very important to establish an efficient method for sensitive determination of trace copper, with regard to the associated health concerns and environmental monitoring. Although conventional analytical techniques including voltammetry and potentiometry,^8^ inductively coupled plasma mass spectroscopy,^9^ atomic absorption spectroscopy/emission spectroscopy,^10^ could meet the demand of sensitive and selective measurement of Cu(ii) in water samples, all these methods are not only time-consuming, labor-intensive, and laboratory-based but also require expensive instrumentation and large sample volume. In this frame, there is a crucial requirement for the development of reliable, fast, and cheap techniques to detect Cu(ii) in water samples.

Recently, various fluorescent sensors including organic dyes, quantum dots (QDs) and carbon dots (CDs), with the festures of simplicity, high sensitivity, good selectivity and rapid response, show great potential in the sensitive detection of Cu(ii). Moreover, fluorescent sensors possess another unparallel advantage, that is, their visualization capability for the determination of analyte with the naked eye by the aid of a simple ultraviolet (UV) lamp. Owing to the classical success of pH test paper, the fluorescent test papers have been widely explored by assembling or printing the fluorescent probes onto a piece of paper-based substrates for the visual assays with low cost, easy operation, and portable feasibility, for example, our group has innovated the color-multiplexing fluorescent test papers by the cooperative employments of QDs and CDs for the visual detections of arsenic ions^11^ and blood sugar,^12^ in which one of two fluorophores were used as the internal color standard to enhance the visualization contrast. The obtained dosage-sensitive color evolution was similar to the performance of pH test paper, but the bad compatibility between QDs and CDs renders the preparation procedure very tedious and the environmental toxicity of QDs also limit the usage of test papers. As is known to all, organic dyes often suffer from fast-photobleaching, low fluorescence quantum yield, narrow excitation spectra.^13,14^ And most QDs are based on semiconductors that contain heavy metals, such as cadmium, and their applications are thus limited for well-known toxicity and high cost.^15–17^ In comparison with the organic dyes and QDs, CDs possess many outstanding advantages, such as low toxicity, biocompatibility, low cost and chemical inertness in addition to having similar fluorescence properties.^18^

Herein, we report a novel dual-emissive fluorescent probe and its as-prepared text paper for the visual detection of Cu(ii). The dual-emission ratiometric fluorescence probe that prepared by mixing blue CDs (BCDs) and orange CDs (OCDs) with fluorescence intensity ratio of 4 : 1 possesses two emission peaks at 440 nm and 610 nm under a single wavelength excitation of 360 nm. The blue fluorescence of the BCDs can be quenched by Cu(ii), while the orange fluorescence of the OCDs is insensitive to the analyte. The control of emissive intensity on the blue and orange fluorescences in the probe allows the color evolution from blue to orange with the concentrations of Cu(ii), which can be conveniently observed by the naked eye under a UV lamp without any complicated instrumentation.

## 2. Experimental section

### 2.1 Reagents and instruments

Citric acid, polyethylenimine (*M* = 600), ethanol and all metal salts were supplied by Sinopharm Chemical Reagent Company, Ltd. (Shanghai, China). *P*-phenylenediamine (*p*-PDA) was purchased from Sigma-Aldrich. All chemicals were used as received without further purification unless otherwise specified. Ultrapure water (18.2 MΩ cm) was produced with a Millipore water purification system.

Fluorescence measurement was recorded on a Perkin-Elmer LS-55 luminescence spectrometer (Liantriant, UK). The structures and morphologies of CDs were examined using a JEOL 2010 transmission electron microscope. The UV-visible absorption spectra were obtained with a Shimadzu UV-2550 spectrometer. Infrared spectra of infrared spectra of the dried CDs *etc.* dispersed in KBr pellets were recorded on a Thermo-Fisher Nicolet iS10 FT-IR spectrometer. Fluorescent photos were taken under AGL-9406 portable UV lamp (254 nm) by a Canon 350 D digital camera.

### 2.2 Synthesis of BCDs

BCDs were synthesized through a simple Hydrothermal method.^19^ 1.0 g citric acid dissolved in 30 mL ultrapure water firstly. Subsequently, 0.5 g polyethylenimine was added to the solution. Finally, the mixed solution was transferred to a poly(tetrafluoroethylene)-lined autoclave, and heated at 200 °C for 6 h. After cooling to room temperature naturally the resultant BCDs were purified by dialysis for 72 h. The purified BCDs were dispersed in ultrapure water (0.03 mg mL^−1^) and stored at 4 °C for further use.

### 2.3 Synthesis of OCDs

OCDs were prepared according to the reported solvothermal method with a minor modification.^20^ 0.3 g of *p*-PDA was dissolved in 30 mL ethanol, and the solution was subsequently transferred into a poly(tetrafluoroethylene)-lined autoclave. After heated at 180 °C for 12 h and then cooled down to room temperature naturally, the mixture was purified through a silica chromatography column using ethyl acetate as eluent. The final product was obtained by drying in a rotary evaporator, and the purified OCDs were redispersed in ultrapure water (0.07 mg mL^−1^) and stored at 4 °C for further use.

### 2.4 Preparation of test papers

Washed the commercial ink cartridge completely with ultrapure water until the ink powder was cleared away, and dried it in an oven at 60 °C for 4 h. Then, the ink replaced by the ratiometric fluorescence probe was injected into the vacant cartridge, and a filter paper was sticked onto a piece of A4 paper. The ratiometric fluorescence probe was printed on the filter paper through an inkjet printer connected with a computer, and the printing process was repeated 10–20 times to enhance the amount of probe. Finally, the filter paper displayed strong blue fluorescent under a 365 nm UV lamp.

### 2.5 Detections of Cu(ii)

Cu(ii) with different concentrations were added to the ratiometric fluorescent probe solution which is prepared by mixing BCDs and OCDs with the fluorescence intensity ratio of 4 : 1 in 2 mL of HEPES buffer (pH = 7.4), and reacted for 3 min. The resulting fluorescence spectra were recorded by a fluorescence spectrometer.

Detections of Cu(ii) on test paper: different concentrations of Cu(ii) dropped onto the as-prepared test paper, and subsequently the color changes of test paper were observed under a 365 nm UV lamp.

### 2.6 Analysis of Cu(ii) in tap water and lake water samples

Detections of Cu(ii) in real water samples including tap water and lake water was performed to evaluate the applicability of the fluorescent probe in environmental samples. Tap water were obtained from our lab and the lake water sample was collected from a local lake. The water samples collected were filtered by ordinary qualitative filter paper and 0.45 μm Supor filters to remove the impurities. Then, different concentrations of Cu(ii) were added into the as-prepared water samples and these samples were analyzed by the fluorescent probes. The average was obtained from three independent measurements and presented with a standard deviation. And the samples were also analyzed by the test paper.

## 3. Results and discussion

**Fig. 1:**
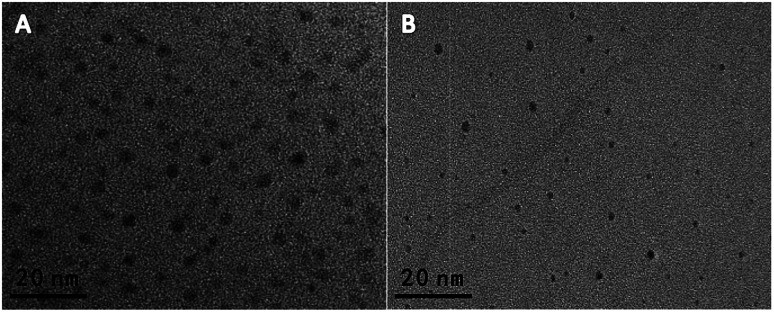
TEM image (A) of BCDs and (B) of OCDs.

The BCDs' properties were characterized by transmission electron microscopy (TEM), UV-vis absorption, FTIR spectroscopy, and fluorescence spectroscopy. As shown in Fig. 1A, the as-prepared BCDs have a good monodispersity with a diameter of ∼5 nm. And, the BCDs present two characteristic absorption at 248 nm and 354 nm, which are ascribed to the large amount of π-electrons in the sp^2^-hybridized structure and the n–π* transition of CDs, respectively (the red curve in Fig. S1A†).^21,22^ The fluorescence spectrum of BCDs exhibited an emission peak at 440 nm with an excitation of 360 nm (the black curve in Fig. S1A†), and a bright blue fluorescence was under a 365 nm UV lamp (inset in Fig. S1A†). Moreover, the FT-IR spectrum was carried out to obtain further structural insights about the BCDs. As shown in Fig. S2A,† the absorption bands at 1376, 1441 and 2944 cm^−1^ are attributed to C–H bending vibrations. Additionally, the peak at 1648 cm^−1^, the strong peak of 1562 cm^−1^, and 1300 cm^−1^ are attributed to amide C=O, N–H, and C–N respectively.^23^ And the absorption band at 3420 cm^−1^ suggests the presence of –NH and/or –OH groups, which is propitious to improve the hydrophilicity of BCDs solution.^19^ As shown in Fig. 1B, the obtained ∼3 nm sized OCDs were highly monodispersive. And, the OCDs exhibited a orange emission centered at 610 nm with the excitation of 360 nm (Fig. S1B†), and a bright orange fluorescence was observed under a 365 nm UV lamp (the inset image of Fig. S1B†). The UV-vis spectrum has two characteristic absorption peaks centered at 239 nm and 281 nm (the red line in Fig. S1B†), and the two peaks are assigned to the π–π* transitions of C=C and C=N bonds in the aromatic rings, respectively, which are identical to those of *p*-PDA.^24–26^ Additionally, in FT-IR spectrum of Fig. S2B,† the two sharp peaks at 1635 and 1515 cm^−1^ ascribed to the C=N and C=C stretching vibrations, respectively, and the peak at 818 cm^−1^ corresponded to the out-plane bending of benzene ring.^20,24,25^

As shown in Fig. S3,† the fluorescence excitation peaks of the BCDs are at 218 nm, 245 nm and 354 nm, and those of the OCDs are at 280, 365 and 484 nm. Moreover, as illustrated in Fig. S4,† the fluorescence intensities of the BCDs shows a tendency to rise first and then decline by adjusting the excitation wavelength from 330 nm to 370 nm. Similarly, the fluorescence intensities of the OCDs shows a same tendency by adjusting the excitation wavelength from 340 nm to 380 nm. So, in order to excite the BCDs and OCDs simultaneously, we choose 360 nm as the excitation band for the ratiometric fluorescence probe. As shown in the fluorescence spectra of the BCDs, OCDs and ratiometric probe (Fig. S5†), the BCDs and OCDs show a maximum emission at 440 and 610 nm, respectively, and the ratiometric probe which disperses well in water can exhibit dual-emission bands at 440 nm and 610 nm under a single wavelength excitation. As shown in Fig. S6,† the stability of the ratiometric probe is systematically investigated by fluorescence spectra in aqueous solution. The fluorescence intensity ratios (*I*_440_/*I*_610_) of the probe remain unchanged over 2 h, demonstrating its excellent photostability.

**Scheme 1:**
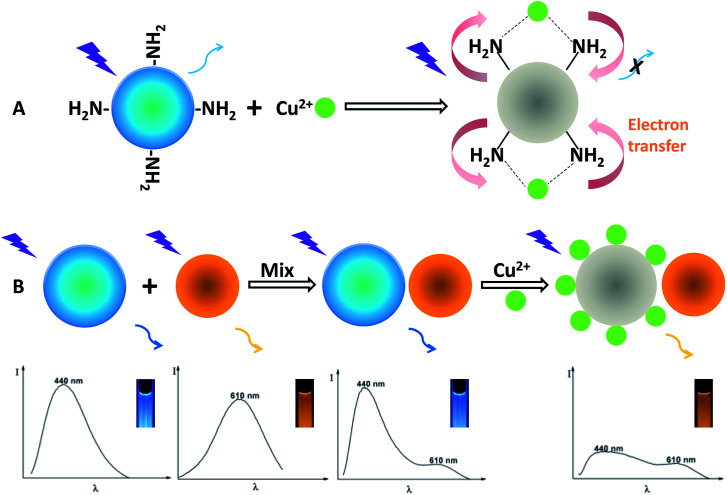
Schematic illustration of the visual detection principle for Cu(ii) and the formation of the dual-emission ratiometric fluorescence probe.

Fig. S7† presents the emission spectra of the OCDs in response to Cu(ii), showing that the fluorescence spectra remain constant without an obvious change of fluorescence color upon the addition of Cu(ii) up to a concentration of 500 nM. Conversely, the fluorescence spectra of the BCDs can be greatly quenched upon gradual addition of Cu(ii) from 0 to 500 nM, but the color change of the single fluorescence quenching of the BCDs is hard to be distinguished by the naked eyes compared with the ratiometric probe. The structure of the dual-emission fluorescent probe and the working principle for visual detection of Cu(ii) are illustrated in Scheme 1. To design the ratiometric fluorescence probe, the BCDs act as a reaction site for Cu(ii), in which the blue fluorescence is effectively quenched by Cu(ii) (Fig. S7A†), and OCDs are selected as the reference signal in the fluorescent probe due to its good photostability and chemical inertness in the presence of Cu(ii) (Fig. S7B†). When Cu(ii) is present, the complexation between Cu(ii) and amino groups of BCDs leads to the splitting of d orbital of Cu(ii). Therefore, electrons in the excited state of BCDs have an opportunity to transfer to the d orbital of Cu(ii). Electron transition in the radiation form (fluorescence emission) of BCDs is consequently restrained, leading to fluorescence quenching.^19^

**Fig. 2:**
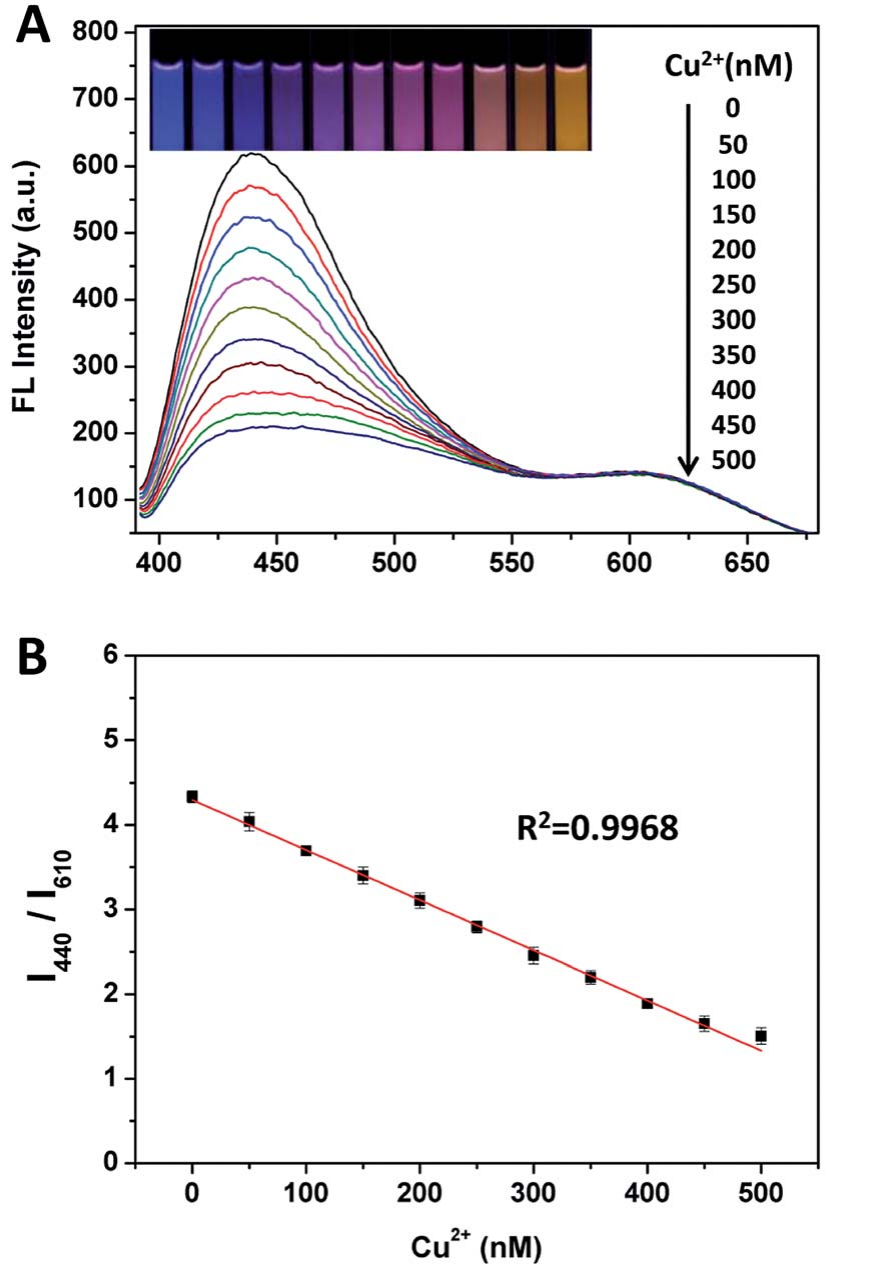
(A) The fluorescent spectra of mixture of BCDs and OCDs at ratios 4 : 1 with the addition of Cu(ii). The inset shows the corresponding fluorescent photo under 365 nm UV lamp. (B) The plot of *I*_440_/*I*_610_ of the ratiometric probe *versus* Cu(ii) concentrations.

We have investigated three different ratio of blue/orange to obtain the widest color variation with the mixture of blue and orange. BCDs and OCDs are mixed and the ratio of emission intensity of blue to orange was adjusted to 4 : 1 (Fig. S8†), which could display a very wide/consecutive luminescence “from blue to orange”. As shown in Fig. 2A, for evaluating the sensitivity of the ratiometric probe, fluorescence responses were measured upon the addition of different amounts of Cu(ii). The slight variation of the intensity ratios of the two emission peaks lead to a distinguishable fluorescence color change from blue to purple, to pink and to orange, which is available for the visual detection of Cu(ii) by the naked eye. Fig. 2B shows that the fluorescence intensity ratio (*I*_440_/*I*_610_) is closely related to the concentration of Cu(ii). To quantitatively evaluate the amounts of Cu(ii), a good linear relationship (*R*^2^ = 0.9968) for the concentration of Cu(ii) ranging from 0 to 500 nM was obtained by plotting the *I*_440_/*I*_610_ ratio *versus* the concentrations of Cu(ii). The detection limit, which was defined as 3 times the standard deviation of background (3*σ*), was calculated to be as low as 7.31 nM. The dynamic experiment demonstrated that the fluorescence response to Cu^2+^ was completed in ∼3 min (Fig. S9†).

**Fig. 3:**
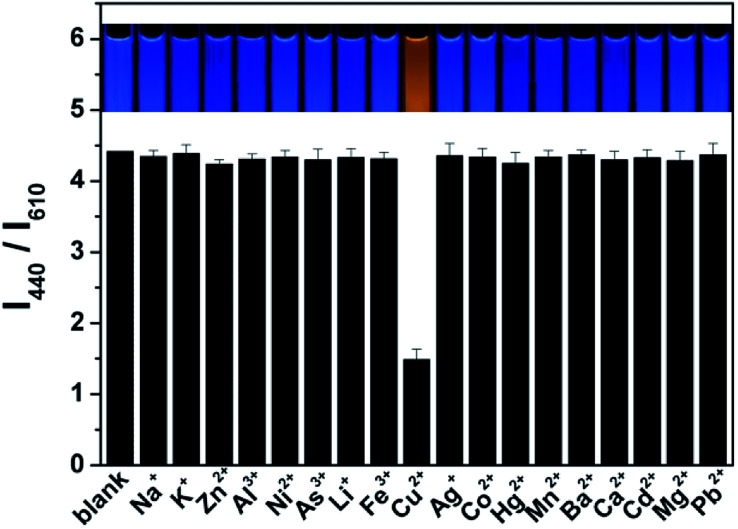
The ratiometric fluorescent responses to various metallic ions and. The selectivity tests were done in HEPES buffer (pH = 7.4) with the addition of 5 μM various metallic ions and 0.5 μM Cu(ii) into the mixing BCDs/OCDs (4 : 1 in fluorescent intensity). The inset images show the corresponding fluorescent photos under a 365 nm UV lamp.

The fluorescent intensity ratio (*I*_380_/*I*_620_) was measured by adding various metal ions in the ratiometric fluorescent probe solution at same conditions to examine the selectivity of the ratiometric fluorescence probe for Cu(ii). As shown in Fig. 3 and S10,† while the *I*_440_/*I*_610_ ratio is quenched about 80% by Cu(ii) at 0.5 μM, no obvious change in *I*_440_/*I*_610_ and fluorescent color was detected with the additions of 5 μM Na^+^, K^+^, Zn^2+^, Al^3+^, Ni^2+^, As^3+^, Li^+^, Fe^3+^, Ag^+^, Co^2+^, Hg^2+^, Mn^2+^, Ba^2+^, Ca^2+^, Cd^2+^, Mg^2+^, and Pb^2+^ into the probe solution. Moreover, the simultaneous addition of 5 μM Na^+^, K^+^, Zn^2+^, Al^3+^, Ni^2+^, As^3+^, Li^+^, Fe^3+^, Ag^+^, Co^2+^, Mn^2+^, Ba^2+^, Ca^2+^, Cd^2+^, Mg^2+^, and Pb^2+^ into the probe solution quenched the *I*_440_/*I*_610_ ratio by only about 20% (Fig. S11†). However, after further addition of 500 nM Cu^2+^, the fluorescence intensity ratio (*I*_440_/*I*_610_) of the probe changes greatly. It should be noted here that, in the complexation reactions with amines, Ag^+^ and Cu^2+^ may have similar reactivities. However, Ag^+^ does not inhibit the fluorescence response of CD, and the presence of Ag^+^ does not affect the quenching activity of Cu^2+^ either, this might result from the formed of silver amine complexes.^27^ It is noted that Hg^2+^ can slightly quench the fluorescence of the probe, but its interference can easily be suppressed by a simple sample pretreatment with KI, NaCl, and rhodamine B.^28^ These data indicate that the ratiometric fluorescent probe exhibits excellent selectivity for Cu(ii).

The feasibility of the ratiometric fluorescent probe for detecting Cu(ii) in real samples was explored by natural water (lake water and tap water). Tap water and lake water were first filtered to remove the undissolved substances, and then spiked with Cu(ii) at the concentrations of 200, 300 and 400 nM. The results are summarized in Table 1. For the three samples, they both have similar results and good recovery rates range from 98.17–104.04%. These results imply that the ratiometric fluorescent probe is likely to be capable of practically useful Cu(ii) detection upon further development.

**Table 1:** The recoveries of Cu(ii) in tap water and lake water by the measurements of ratiometric fluorescence

Spiked concentration (nM)	Tap water			Lake water		
	Found (nM)	Recovery (%)	RSD (%)	Found (nM)	Recovery (%)	RSD (%)
200	206.2739	103.14	4.92	205.0922	102.55	5.10
300	308.5576	102.85	4.77	312.1122	104.04	5.32
400	392.6911	98.17	3.13	397.8657	99.47	4.61

**Fig. 4:**
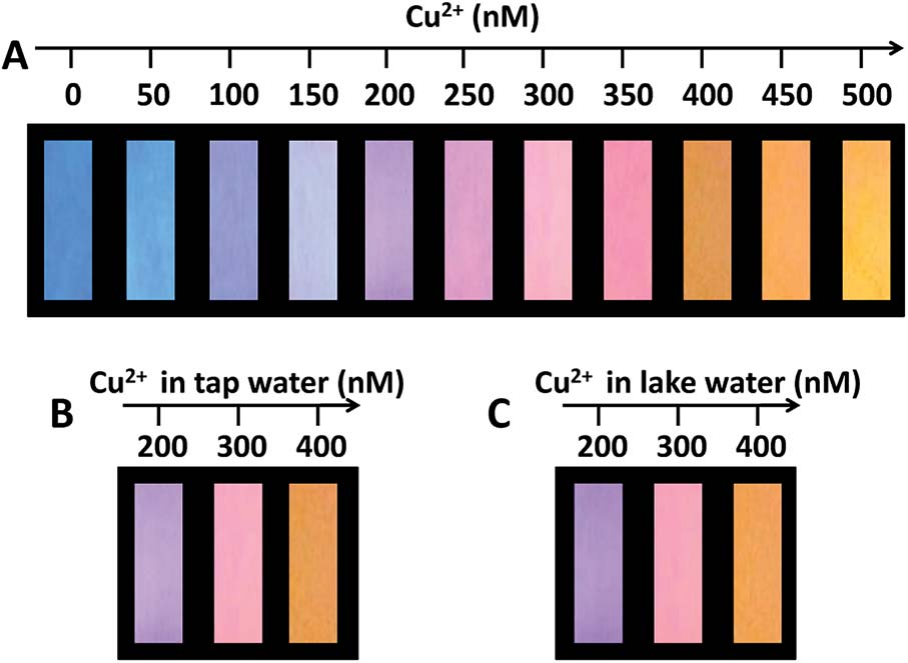
(A) The visualization detection of Cu(ii) using the fluorescent test papers prepared by printing dual colored CDs ink onto a piece of filter paper. (B and C) The visual detections of Cu(ii) in tap water and lake water, respectively. The photos were taken under a 365 nm UV lamp.

Following the above strategy, we have prepared fluorescent test paper for the visual semiquantification detection of Cu(ii). The BCDs and OCDs were mixed and adjusted to a ratiometric fluorescent intensity of 4 : 1, and the aqueous mixture was used as fluorescent ink. The test papers with the fluorescent ink displayed a highly uniform blue brightness on the whole piece of paper under the irradiation of UV lamp at 365 nm. As shown in Fig. 4A, while dropping the aqueous solution of Cu(ii) onto the test paper, the colors of test papers under UV lamp consecutively and gradually evolved from blue to purple to pink and to orange with the increment of Cu(ii) concentration from 0 to 500 nM. Fig. S12† also shows that the temporal color evolution with the addition of 500 nM Cu(ii) displayed a serial of intermediate colors from blue to orange in 4 min. We further examined the applicability of the fluorescent test papers for the detection of Cu(ii) in real water samples. The fluorescent test papers gave out the obvious color responses to the different Cu(ii) concentrations, and the corresponding colors are identical in the cases of tap water and lake water (Fig. 4B and C). Moreover, the tendency of color evolutions for the detection of real samples is very similar to that in Fig. 4A. The excellent visual effect and accuracy suggest that the fluorescent test papers can meet with the requirements for the visual detection of Cu(ii) in water samples.

## 4. Conclusion

In summary, we have designed a dual-emissive fluorescent probe prepared by mixing BCDs and OCDs with fluorescence intensity ratio of 4 : 1 and its as-prepared text paper for the visual detection of Cu(ii). With the addition of Cu(ii), the variation of fluorescence intensity ratios produce an obvious change of the fluorescence color from blue to orange, which can be conveniently observed by the naked eye under a UV lamp without any complicated instrumentation. The detection limit of this probe reaches as low as 7.31 nM, which is much lower than the maximum level of Cu(ii) (20 μM) in drinking water (EPA). The probe exhibits enhanced sensitivity and reliability of visual detection compared with single CDs-based probes, and possess many outstanding advantages, such as low toxicity, biocompatibility, low cost. Furthermore, we apply the ratiometric probe for visual identification of Cu(ii) in real water samples. The results reported here prospect the bright future of fluorescent test paper for the feasible and inexpensive applications in chemical and biological sensing.

## Conflicts of interest

There are no conflicts to declare.

## Supplementary Material

RA-008-C8RA00917A-s001
