# Supplementary material for: Semi-quantitative and visual assay of copper ions by fluorescent test paper constructed with dual-emission carbon dots
Source: RSC Adv. 2018 Apr 3;8(23):12708–13. doi: 10.1039/c8ra00917a (PMC9079620; doi:10.1039/c8ra00917a)
Supplement: RA-008-C8RA00917A-s001 [file RA-008-C8RA00917A-s001.pdf]

Electronic Supplementary Material (ESI) for RSC Advances.
This journal is © The Royal Society of Chemistry 2018

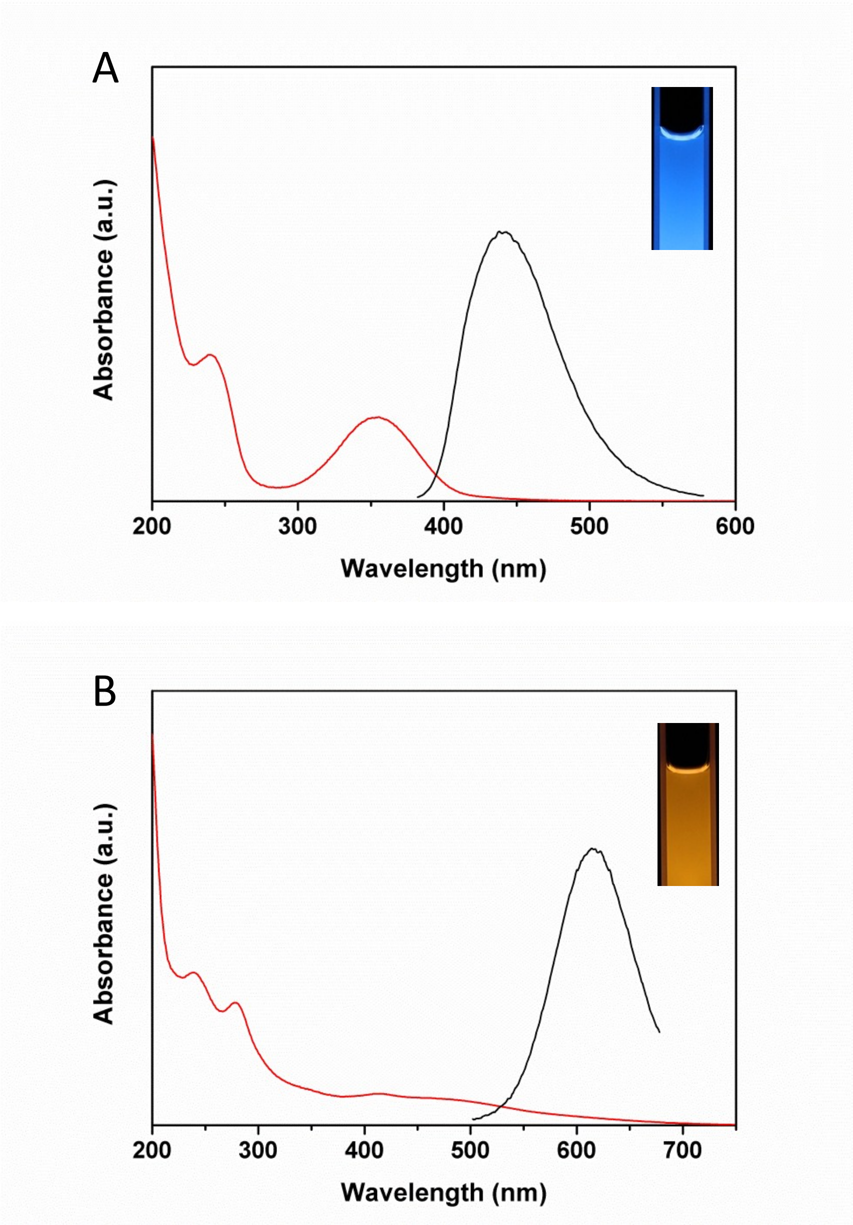

Figure S1. (A) UV-vis absorbance and fluorescent emission of BCDs (The inset show the corresponding color under a 365 nm UV lamp). (B) UV-vis absorbance and fluorescent emission of OCDs (The inset show the corresponding color under a 365 nm UV lamp).

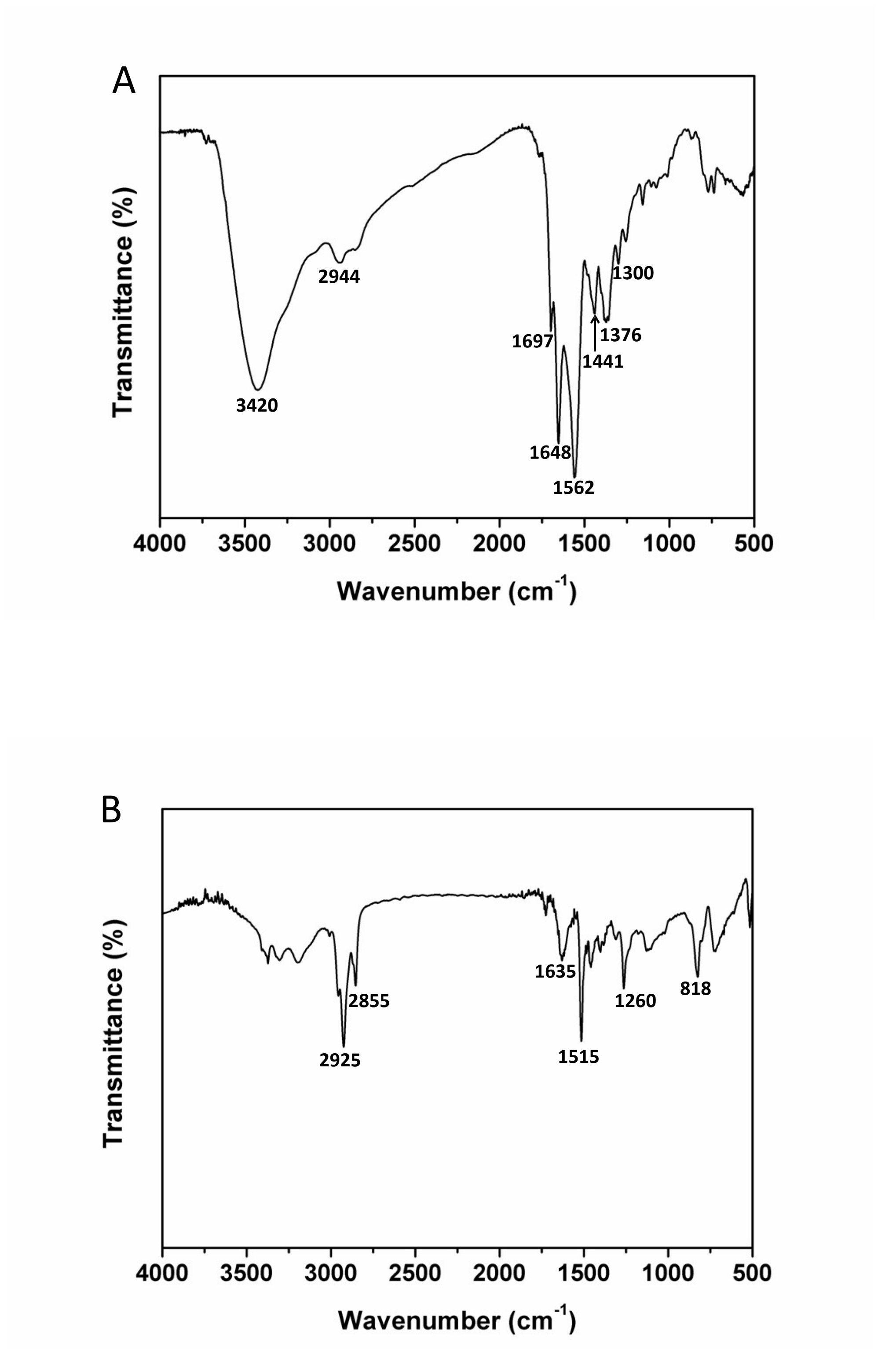

Figure S2. The FT-IR spectra (A) of BCDs and (B) of OCDs.

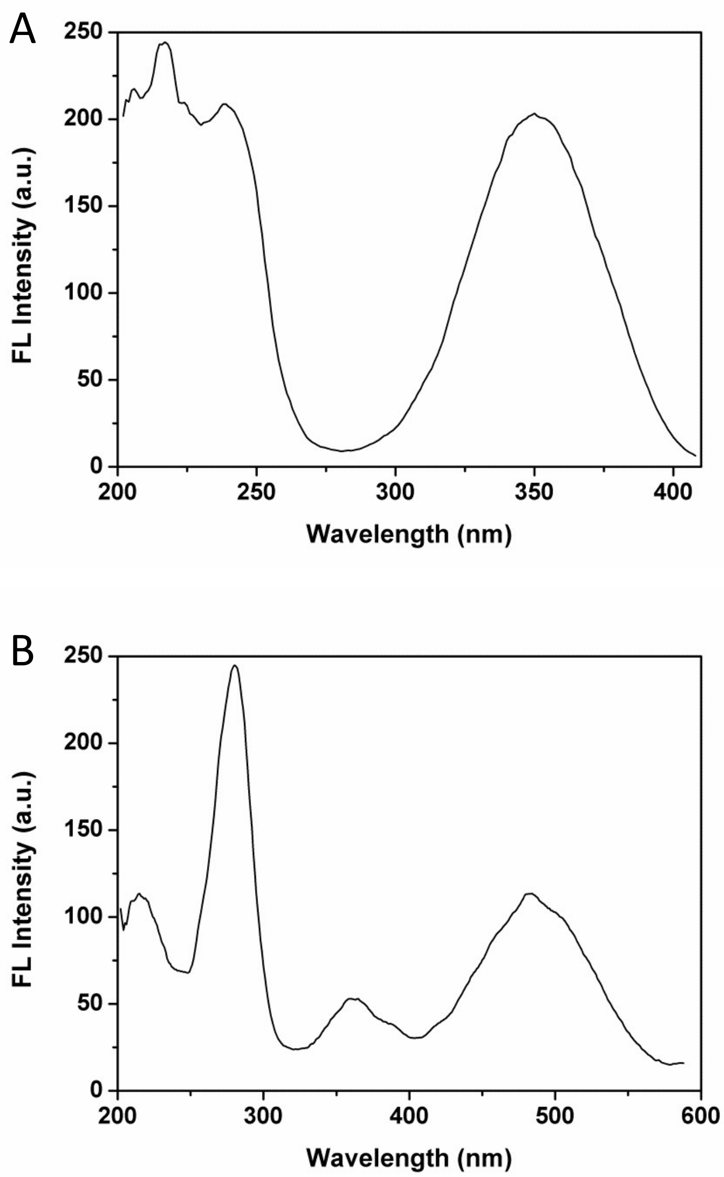

Figure S3. The excitation spectrum (A) of BCDs and (B) of OCDs.

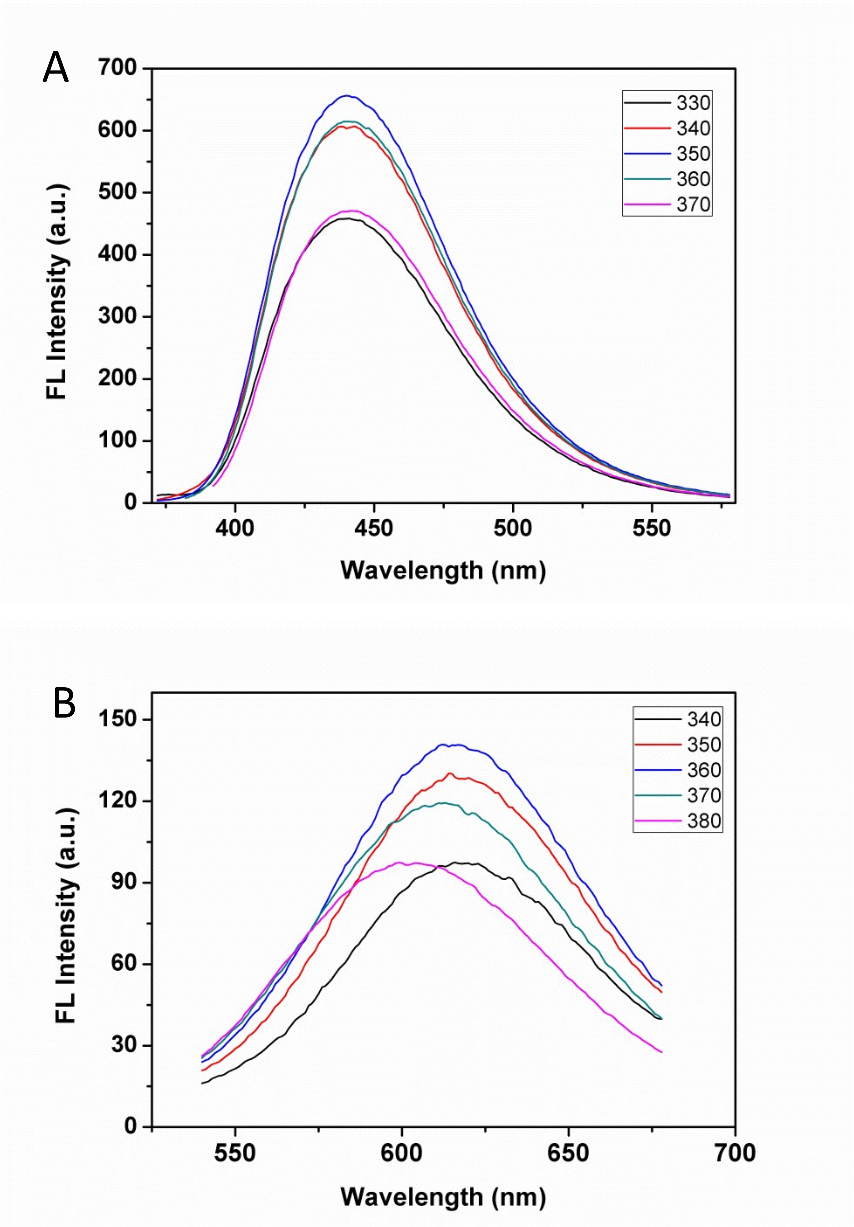

Figure S4. (A) FL spectra of the BCDs excited at different wavelengths, (B) FL spectra of the OCDs excited at different wavelengths.

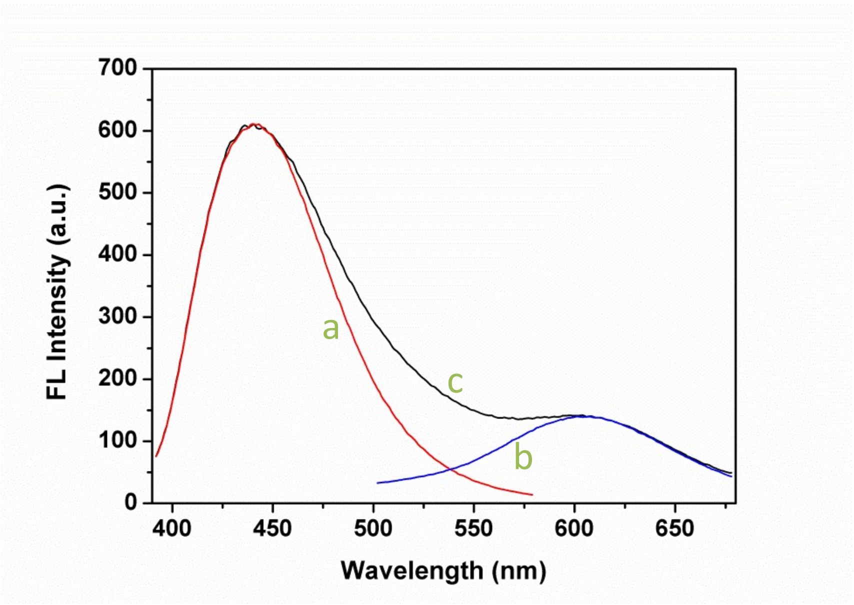

Figure S5. Fluorescence emission spectra (λ_{ex} = 360 nm) of (a) BCDs, (b) OCDs and (c) mixing BCDs and OCDs, respectively.

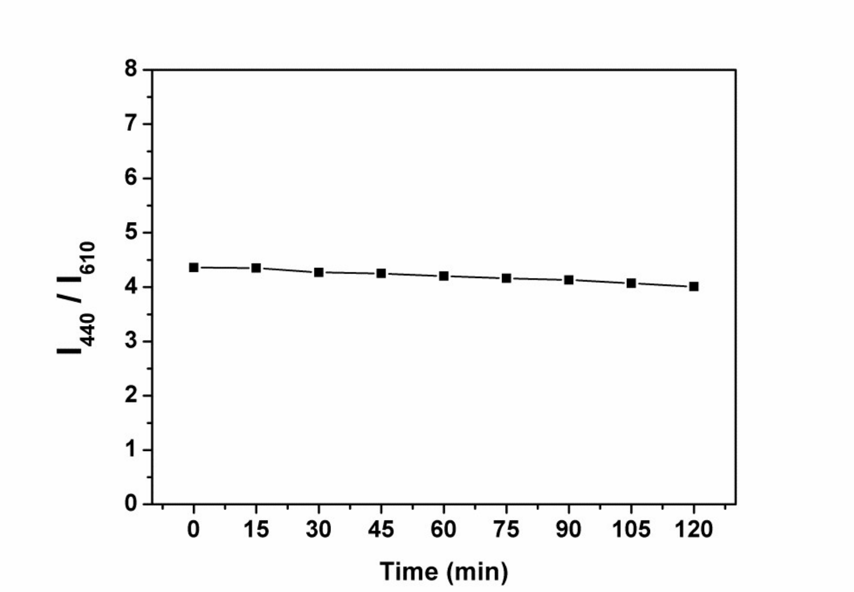

Figure S6. Photostability of the ratiometric fluorescence I_{440}/I_{610} of mixing BCDs/OCDs with the time exposed to 365 nm ultraviolet light for 15 min each time (I_{440} and I_{610} are the fluorescence intensities of BCDs and OCDs, respectively).

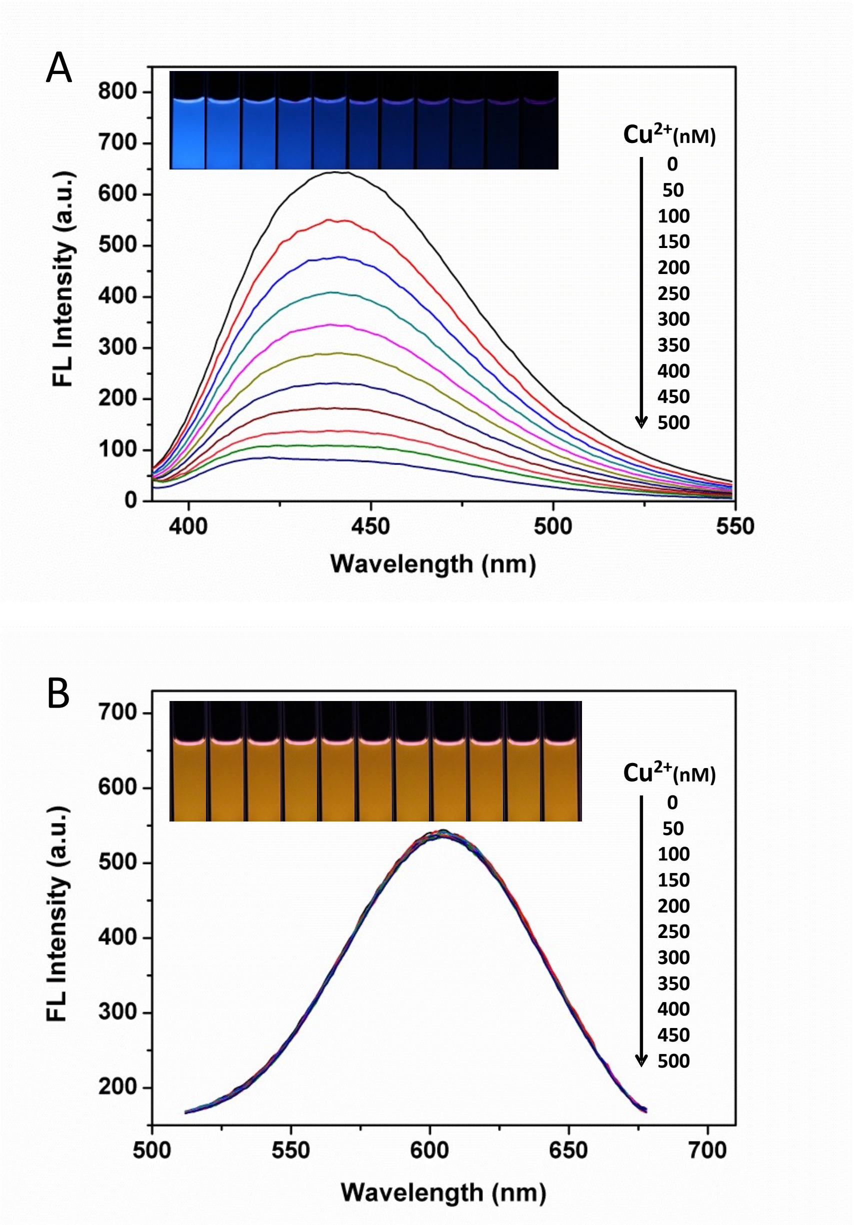

Figure S7. The fluorescent emission spectra (λ_{ex}= 360 nm) of (A) BCDs and (B) OCDs with the addition of Cu^{2+}. The inset photos show the corresponding color evolutions under a 365 nm UV lamp.

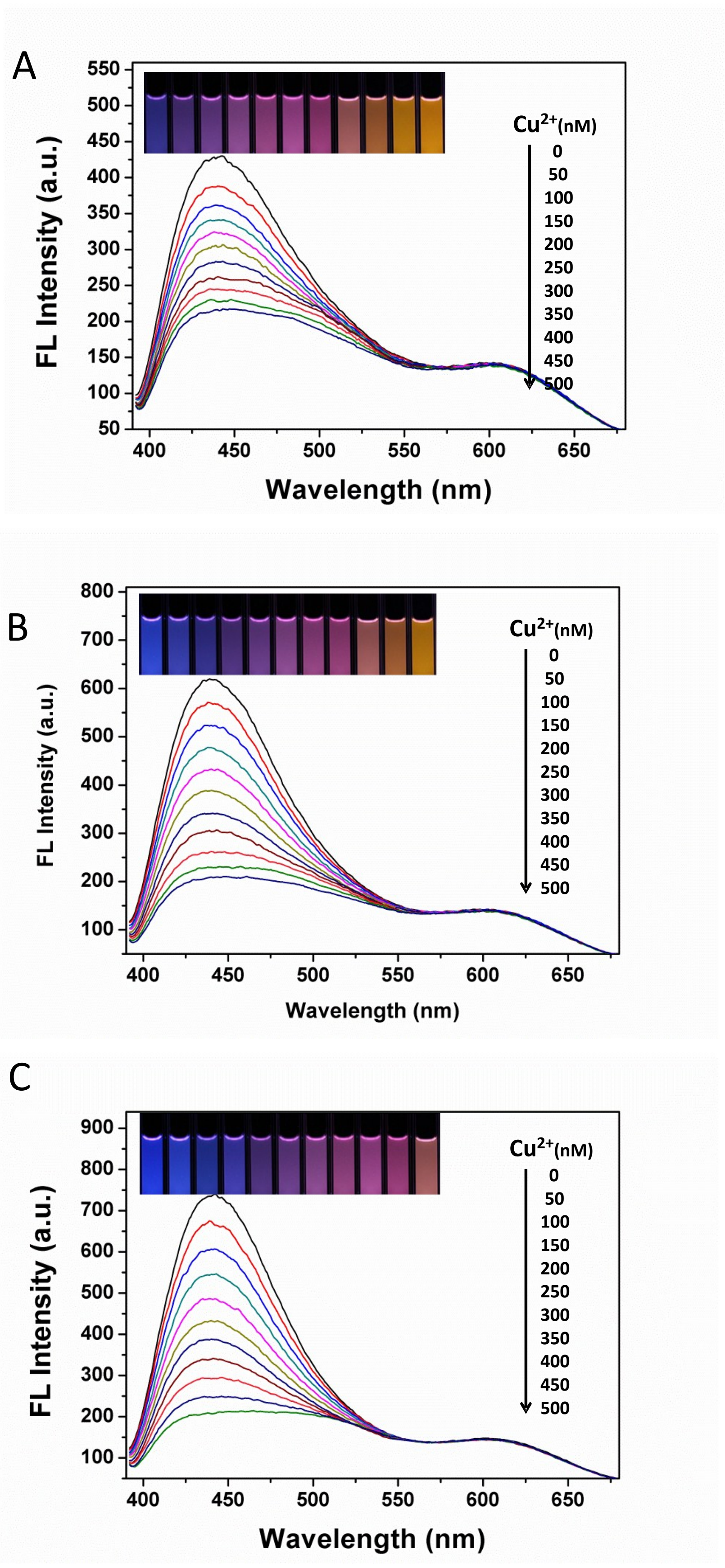

Figure S8. The fluorescent spectra of the mixing BCDs/OCDs with the addition of Cu^{2+} ions. Before measurements, the fluorescent intensity ratios in the mixing BCDs/OCDs

were adjusted to (A) 3:1, (B) 4:1, (C) 5:1. The insets show the corresponding fluorescent photos under a 365 nm UV lamp.

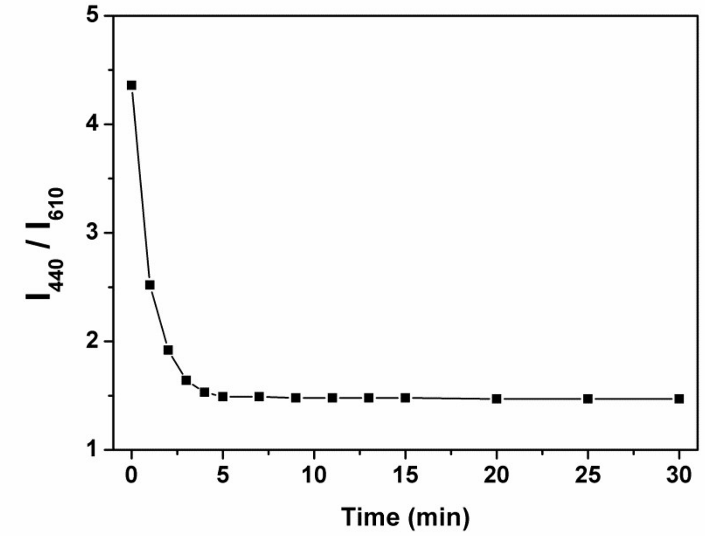

Figure S9. The temporal fluorescent response by the ratiomertric fluorescence I_{440}/I_{610} after the addition of 500 nM Cu^{2+}.

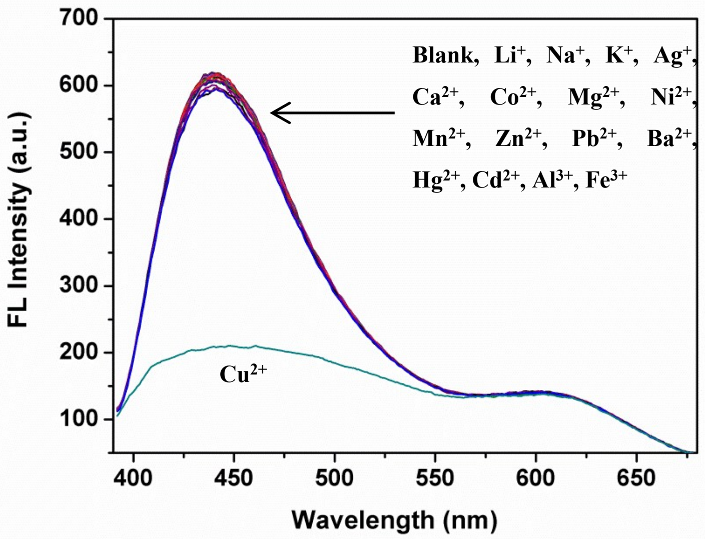

Figure S10. The ratiometric fluorescent responses to various metallic ions with Cu^{2+}. The selectivity tests were done in HEPES buffer (pH=7.4) with the addition of 5 μM various metallic ions into the mixing BCDs/OCDs (4:1 in fluorescent intensity).

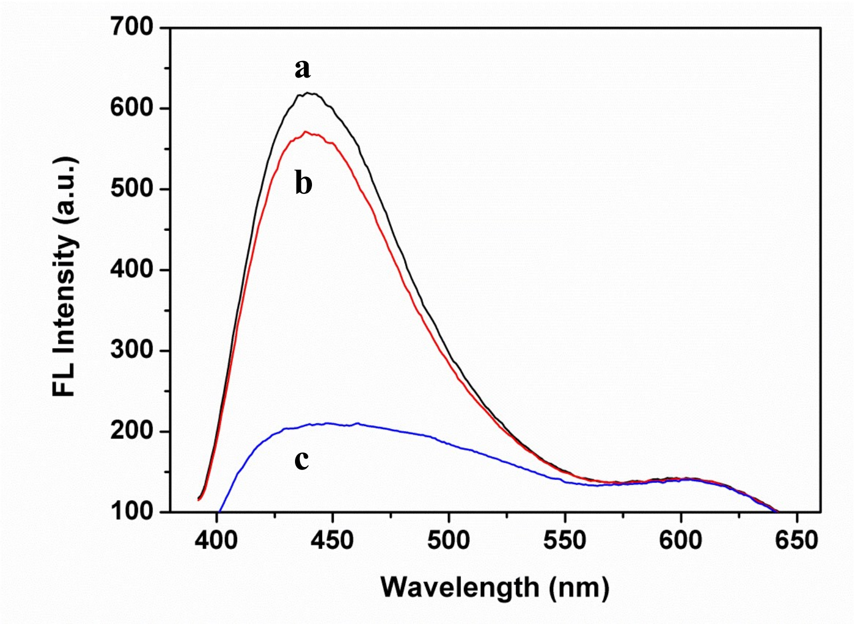

Figure S11. The fluorescent spectra of the ratiometric probe after and before the additions of metallic ions. (a) Without any addition of metallic ions. (b) The addition of 5 μM Na^+, K^+, Zn^{2+}, Al^{3+}, Ni^{2+}, As^{3+}, Li^+, Fe^{3+}, Ag^+, Co^{2+}, Hg^{2+}, Mn^{2+}, Ba^{2+}, Ca^{2+}, Cd^{2+}, Mg^{2+}, and Pb^{2+} together. (c) A subsequent addition of 500 nM Cu^{2+} in (b).

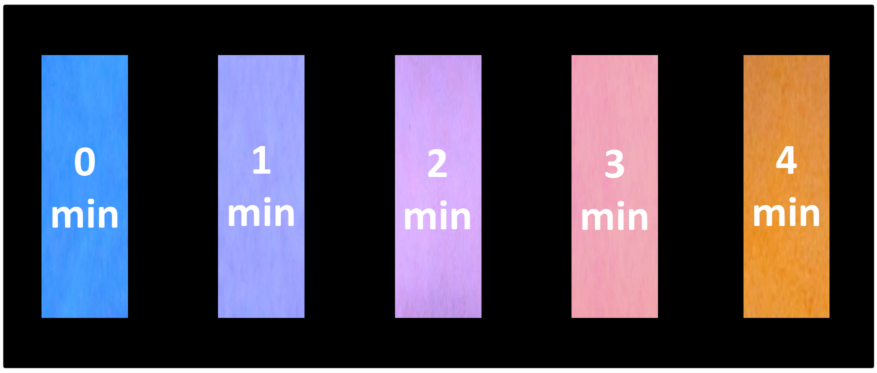

Figure S12. The temporal color changes of fluorescent test paper upon the addition of 500 nM Cu^{2+}. The photos were taken under a 365 nm UV lamp.
